# How digital finance affects income distribution: Evidence from 280 cities in China

**DOI:** 10.1371/journal.pone.0267486

**Published:** 2022-05-18

**Authors:** Jiang Youxue, Shimei

**Affiliations:** 1 School of Finance, Jiangsu Vocational Institute of Commerce, Nanjing, China; 2 Business School, Southeastern University, Nanjing, China; Universiti Malaysia Sabah, MALAYSIA

## Abstract

This paper empirically tests the relationship between digital finance and income distribution of residents in 280 cities in China from 2011 to 2020 using linear and nonlinear models, respectively. Based on the Greenwood-Jovanovic (G-J) theory of output grow, the empirical study shows that there is a Kuznets effect of digital finance development on the income distribution of Chinese residents, and most regions have not yet crossed the inflection point of the bell-shaped curve, and the income gap within regions will continue to increase with the development of digital finance. Furthermore, the threshold model test shows that the positive effect of digital finance on the income disparity of residents may initially increase with the increase of regional economic level. However, when the regional economic development reaches a higher stage, the negative effect of digital finance development on the income distribution of residents will be significantly reduced.

## I. Introduction

Emerging digital technology innovations are increasingly widely applied. The rapid development of information technology represented by big data and artificial intelligence drives continuous optimization and innovation in the financial industry [1,2]. In recent years, with the continuous empowerment of digital technology, the subjects of digital financial services in China are becoming more and more diversified, the construction of essential communication is becoming more and more complete, and digital finance is becoming an important direction to promote policy implementation and achieve sustainable development of inclusive finance [3,4].

As a product of the combination of digital technology and traditional finance, digital finance necessarily has financial attributes that no longer rely on the physical channels on which traditional finance relies, are more penetrating at the geographic level, and have lower cost advantages [5,6]. Based on theories of financial development and income distribution, we find that academics seek not only consensus but also exploration and extension of the issue, but all acknowledge the critical role played by digital finance in income distribution [3,7,8]. In the era of the digital economy, digital finance has become an essential engine of financial development everywhere. It plays a vital role in both inter-regional development and intra-regional development. There are different views on the impact of digital finance development on the current income distribution in China. Some literature argues that the inclusive nature of digital finance has significantly reduced the gap in income distribution, that access to payments, credit, and digital financial services in rural areas will significantly contribute to inclusive growth within regions, and that digital payment instruments can effectively reduce the current urban-rural income gap in China [9,10]. And digital finance has more than a simple linear impact on the income distribution of the population, there are some nonlinear characteristics such as threshold characteristics [11]. The existence of threshold effects makes the development of digital finance have different effects on income distribution in different regions and financial exclusion due to the threshold of digital technologies that promote the development of inclusive finance, such as infrastructure, practical application, and institutional environment [12,13]. The integration of inclusive finance and digital technologies is insufficient, and low-income groups still face multiple difficulties accessing essential financial services. We find that low-income people tend to have less education and limited knowledge of finance and the Internet and that digital finance relies on electronic devices. A significant proportion of low-income groups do not have access to appropriate electronic devices or knowledge of the Internet, resulting in unequal access to digital financial applications [14,15]. A concept that must be mentioned here is the digital divide. The inequality in income and education levels of different population segments implies a gap in their access to and information processing. The digital divide will exacerbate the information blockade of the information-poor groups, limiting their opportunities and ways to generate income and creating a new type of stratification. Thus, the digital divide is essentially economic [16,17].

It is easy to find that the literature systematically demonstrating the relationship between digital finance development and income distribution in China is limited, and theoretical and empirical studies still need to be further explored. This paper measures the Gini coefficient of income of each prefecture-level city in China based on the existing literature. It discusses and analyzes the impact of digital finance development on income distribution in China in the context of the current situation of digital finance development in China [18,19]. Unlike previous studies, this paper uses statistical data from 280 prefecture-level cities across China to investigate the link between digital finance development and income disparity in China from a theoretical and empirical perspective. It proposes corresponding policy recommendations based on the findings.

Compared with existing studies, this paper may have the following innovations: most of the existing literature on digital finance and income distribution discusses the linear relationship between the two, with the majority of such literature being representative [3,4,20,21], which have explored whether digital financial development is conducive to raising local income levels and whether there is heterogeneity in the extent to which income levels are raised [10,22,23]. In contrast, some representative literature advocates another view that the relationship between digital finance development and income distribution may have non-linear characteristics, which is less represented in the literature [8,10], where they use threshold effect analysis to explore whether there is a threshold value for digital finance to be helpful in raising residents’ income, who used threshold effects analysis to explore whether there is a threshold for digital finance to contribute to higher income. However, considering the current situation of digital finance development and income distribution in China and the related mechanism of digital finance development on income distribution, it is reasonable to suspect that the two are linearly related and probably have a more complex inner connection. Therefore, based on the linear analysis, this paper adopts two nonlinear methods to verify the Kuznets effect and the threshold characteristics of digital financial development affecting the income distribution of residents, to explore the relationship between digital financial development and the current income gap more comprehensively, and to explain the current phenomenon of "digital divide" in China.

In addition, the current literature on income disparity mainly focuses on the urban-rural level. At the same time, the Gini coefficient measures the overall regional income disparity, and there is almost no research on the relationship between digital finance and overall regional income distribution. This paper uses the most common international Gini coefficient indicator to measure the degree of regional income distribution imbalance, to explore the impact of current digital finance development on China’s income distribution, and to fill the gap in the current research on the relationship between digital finance and overall income distribution in China.

## II. Literature review

### 1. Study on the relationship between digital finance and income distribution

In contemporary economics research, Kuznets was the first to systematically explore the relationship between income disparity and economic growth. [24,25] linked income inequality with economic development and proposed the classic Kuznets curve in economics. He argued that the socio-economic level, measured by per capita income, initially increases in parallel with the income gap. However, as the socio-economic development continues, more people enjoy the fruits of social development, and eventually, the income will tend to equalize. [26] widely applied the Kuznets curve and proposed the classical two-sector model, which argued that the differentiation of enterprises with different productivity would cause income inequality, but labor would become a scarce resource when the economy developed to a particular stage. The inequality of income distribution would gradually decline.

[3] used the Peking University Digital Inclusive Finance Index aimed to explore the mechanism of the current digital inclusive policies implemented in China on the income distribution of urban and rural residents, and found that the development and application of digital technology dramatically improve the current income of residents. Its effect on the income increase of urban residents is more prominent and has a heterogeneous impact on the income increase of different groups in urban and rural areas [20]. The study found that the implementation and development of regional digital finance can significantly increase the income level of residents. [23] used panel data at the provincial level in China from 2011–2015 and confirmed through an econometric model that the current digital inclusive finance in China has positive implications for narrowing the income gap between urban and rural areas within regions [3] compiled relevant data for China during 2004–2014 and applied the VECM model to find that digital financial development can effectively promote intra-regional inclusive finance in China. The increasing level of inclusive financial development has contributed significantly to balanced income distribution in China [22,27] argue that the digitalization of traditional industries only benefits the wealthy class in the industry, which will lead to more significant class differentiation [28] suggest that the "digital divide" between countries and regions around the world is a challenge that governments at all levels must face as digital technologies continue to evolve [29] explored the impact of digital technology on income inequality among residents of 54 countries from 2010 to 2015 and showed that financialization and digital technology widened the gap in income inequality.

As can be seen, many scholars have pointed out that the development of digital technology is likely to widen the gap in income distribution from its original base. However, other scholars such as [30,31] use relevant data from less developed countries to demonstrate that the coverage of mobile networks can help markets function better and improve the welfare of low-income people. [21] delve into the two different phenomena of the digital divide and digital dividend in the Internet market and find that the continuous development of digital platforms can allow people to cross the original digital divide and realize the digital dividend.

### 2. The path mechanism of digital finance affecting income distribution

Employment level: during the development of digital finance, rural residents’ access to credit has been broadened, and using the Internet; rural residents can start their businesses at a lower cost than before; the development of digital finance has increased the feasibility of entrepreneurship in rural groups to some extent, significantly alleviating the poverty-causing nature of farmers, and farmers’ income has increased as a result. Many scholars have focused on the profound impact that the development of digital finance has on the employment of the population, (Dean et al., 2017) focused on the relationship between female entrepreneurship and internet use, it has been proven that the use of the Internet has contributed significantly to the overall employment of women groups and that the use of the Internet can effectively help married, low educated, agricultural households women to access employment opportunities [32] verified the effect of internet use on individual employee behavior and employment quality using a multivariate Probit model and found that internet use significantly increases the likelihood of individuals to engage in standard employment and opportunity entrepreneurship but has the potential to widen the income gap within the region due to the low internet use skills of the poor as well as disadvantaged groups [33] studied the impact of regional Internet availability on local productive life using national data and concluded that intra-regional Internet penetration could effectively increase local employment levels. This effect is more pronounced in less developed rural areas [34], using county-level data from the United States, household access to the Internet significantly increased their probability of employment. This effect was also more pronounced in rural and remote areas.

Other scholars have argued that the mass use of smart devices in the digital economy will impact the employment of low-skilled workers and may lead to the marginalization of low-skilled workers, which will result in a widening of the income distribution gap [35] demonstrated through an empirical study that the increase in the use of industrial machines between 1990 and 2007 was an important reason for the deterioration of the local labor job market in the U.S. The number of machine applications is closely related to the corresponding salaries of workers. An increase in their number will lead to a decrease in the average salary of workers [36] also points out the negative impact of intelligence on the low-skilled labor force. While [37] argue that emerging technologies such as artificial intelligence and machine learning do not significantly affect the current division of labor and labor market.

Credit level: Digital financial inclusion, which has been continuously landed during the development of digital finance, has dramatically eased the credit constraints of residents in rural areas and the financing constraints of small enterprises, which is conducive to widening the employment channels of low-income groups. Therefore many scholars have explored the mechanism of the Internet’s effect on income distribution about its impact on credit constraints. [38] empirically verified the effect of digital finance on the credit constraint of farmers’ groups and proved the positive driving effect of digital finance on the entrepreneurship of rural groups. [34] confirmed that digital finance significantly alleviates the credit constraints faced by households in general and found through heterogeneity analysis that it has more significant consumption incentives for households within less developed regions [39] argued that crowdfunding by digital finance can complement traditional entrepreneurial financing methods.

As the connection between digital finance and residents’ productive life becomes closer, researchers outside China have started to focus on studying the issues related to digital finance and income distribution. The current consensus is that digital finance development can raise urban and rural residents per capita disposable income. However, the level of income increase for different groups of people is not the same, which leads to the consideration of the impact on the income distribution gap. Currently, academics hold the following three views on the relationship between digital finance development and income distribution: The first type is that digital finance development will reduce the income gap. This view is usually based on the inclusive nature of digital finance, which is believed to have a more substantial effect on raising the income of rural people and low-income groups, thus significantly reducing the income gap between urban and rural residents. The second type of view is that the development of digital finance will increase the income gap. This view is mainly found in foreign literature, believing that digital finance is more beneficial to the affluent class. As a result, the "digital divide" will appear between low-income and high-income groups due to the existence of digital technology, which requires our vigilance. The third type of view is that the relationship between the development of digital finance and the income gap shows a Kuznets effect.

The critical impact of digital finance development on the current income distribution has become a consensus among academics, so this paper conducts a theoretical and empirical study on the real issue of digital finance and income distribution based on previous studies. The depth of digital finance use and the regional Gini coefficient is used to measure the development of digital finance and the regional income distribution, respectively, to conduct a comprehensive analysis of the current situation of digital finance development and income inequality of residents, and to analyze the inner mechanism of the actual situation. Based on the data of prefecture-level cities, we analyze the nonlinear relationship between digital finance development and income inequality, and introduce the threshold model to analyze the threshold effect of digital finance development in different regional economic development levels, and refine the effect of its role in different economic levels, which is closer to the current national situation and more accurate guidance for national policy formulation.

## III. Method

### 1. Variable selection

This paper uses panel data for 280 prefecture-level cities in China during 2011–2020 and involves the following variables.

1. **Income gap (GINI)**. As the most widely used indicator to measure regional income imbalance in the international arena, the Gini coefficient mainly indicates the relative deviation of the average income inequality from the overall expectation based on the population distribution. Because the Gini coefficient is complicated to measure, most empirical articles on income disparity in China use the urban-rural income disparity index to measure regional income distribution. In contrast, the urban-rural income disparity index cannot reflect the level of income disparity within urban and rural areas and is one-sided; the Gini coefficient is currently an internationally recognized authoritative index that can measure the imbalance of income distribution in a country or region. Therefore, we use. Therefore, we use the statistical yearbook of each prefecture-level city to measure the Gini coefficient by dividing the income into five equal groups, and the larger the GINI value represents, the greater the degree of unbalanced income distribution within the region. We derive the Gini coefficient for each region of prefecture-level cities based on Eq 1.
$$G=1-\frac{1}{PW}\sum_{i=1}^{n}\left(W_{i-1}+W_{i}\right)\times P_{i}$$ (1)
  Where: *P*_*i*_ represents the total population of the area; *W* represents the total income of the population; *W*_*i*_ represents the cumulative income sum to group *i*.
  In this paper, the Gini coefficients of urban and rural incomes of 280 prefectures in 2011–2020 are calculated using Eq 1. The "group weighting method" proposed by [40] is applied to calculate the Gini coefficients of overall incomes of each prefecture. The following is the formula of the "group weighting method."
$$G=P_{c}^{2}\frac{u_{c}}{u}G_{c}+P_{r}^{2}\frac{u_{r}}{u}G_{r}+P_{c}P_{r}\frac{u_{c}-u_{r}}{u}$$ (2)
  Where: "2" represents the proportion of the urban population to the total population; "3" represents the per capita income of urban residents; "4" represents the Gini coefficient of urban residents’ income of each prefecture-level city; "5" represents the proportion of the rural population to the total population; "6" represents the per capita income of rural residents; "7" represents the Gini coefficient of rural residents’ income of each prefecture-level city; "8" represents the overall per capita income of each prefecture-level city.
  Using Eq 2, this paper calculates the Gini coefficient of the income of all residents in 280 prefecture-level cities from 2011 to 2020.
2. **Digital financial development level (DE)**. In order to accurately portray the development status of digital finance, we must establish scientific indicators to measure the degree of development of digital finance within a region. At this point, it is not accurate to consider only the breadth of digital account coverage in a region and the degree of deepening of the use of digital finance in each region to more accurately portray the development of digital finance. In this paper, we use the depth of use index in the Digital Inclusive Finance Index jointly compiled by the Digital Finance Research Center of Peking University and Ant Financial Services Group to describe the development of digital finance in each region of China. The study contains a comprehensive description of China’s digital finance development and its evolution and has been used in much literature on digital finance.
  The index is based on data from Ant Financial Services’ transaction account data, a comprehensive measure of the current development of digital finance in China at all levels, and has strong credibility. Moreover, its educational value is becoming more and more apparent as the number of people using the data for application research increases. Within this index system, the Digital Inclusive Finance Index contains three major dimensions: breadth of coverage, depth of use and digitalization. This paper measures the development of digital finance in China by using the depth of use, which is a secondary indicator in the Chinese digital inclusive finance system. This indicator is used for the following reasons: First, the three sub-indicators of breadth of coverage, depth of use and digitalization are independent of each other, and the depth of use indicator is the most important indicator and the main indicator compiled by the group, while the other two indicators are subsidiary products of this indicator. Secondly, the depth of use indicator covers six business modules, namely payment, credit, insurance, investment, money fund and credit, which summarize all aspects of digital finance in modern economic and social development, while the other two indicators cover a thin scope and cannot effectively measure the development of digital finance, thus we believe that the depth of use indicator in a region can well represent the development of digital finance in that region.
3. **Control variables**. Referring to previous literature [3,40–42], we chose the level of economic development (GDP), foreign direct investment (FDI), level of openness to the outside world (open), level of urbanization (urban), Internet penetration rate (user), level of financial development (FD), and level of education (Edu) as control variables.

### 2. Descriptive statistics

Considering the availability and consistency of data, this paper takes 2011–2020 as the sample period. It excludes missing samples, covering a total of 280 prefecture-level cities in 28 provinces in China, including the digital financial development index from the Digital Inclusive Finance Development Report compiled by Peking University, and the data required for other variables from the China City Statistical Yearbook, the provincial and prefecture-level city statistical yearbooks, the prefecture-level city Government Work Report" and "Statistical Bulletin of National Economic and Social Development" of each prefecture-level city, etc. This paper uses STATA15.1 software for regression analysis, and the descriptive statistics of the main variables of interest are given in Table 1 below.

**Table 1:** Descriptive statistics of the main variables.

Variable Name	Meaning of variables	Number of observations	Average value	Standard deviation	Minimum value	Maximum value
Gini coefficient (GINI)	Measuring the level of income disparity of prefecture-level cities	4900	0.473	0.053	0.163	0.717
Digital financial development level (DE)	Measuring the degree of digital finance development in each region	4900	151.110	65.110	4.29	325.679
Economic development primary term (GDP)	GDP per capita	4900	4.958	3.539	0.534	53.8722
Foreign direct investment (FDI)	Amount of foreign direct investment in prefecture-level cities/GDP	4900	0.289	0.352	0	3.864
Level of external openness (open)	Total import and export trade of prefecture-level cities/GDP	4900	0.234	0.659	0	9.261
Urbanization (urb)	The urban resident population of prefecture-level cities/total population of prefecture-level cities	4900	55.090	14.521	0.41	100
Internet penetration rate (user)	Internet users in prefecture-level cities/total population of prefecture-level cities	4900	1.018	0.801	0.249	9.614
Financial development level (fd)	The balance of deposits and loans of financial institutions in prefecture-level cities/GDP	4900	2.584	1.875	0	27.327
Level of numerical education (Edu)	Number of general primary and secondary school students in prefecture-level cities/total population of prefecture-level cities	4900	0.120	0.39	0.051	0.517

### 3. Model construction

The linear relationship model between digital financial development and income distribution of the population is constructed as follows.

$$GINI_{it}=\beta_{0}+\beta_{1}*DE_{it}+\beta_{2}CV_{it}+\mu_{i}+\varepsilon_{it}$$ (3)

where: i and t represent each prefecture-level city and year, respectively; *DE*_*it*_" represents the level of digital financial development of prefecture-level city i in year t; *CV* represents a set of control variables affecting the income gap in the region; *μ*_*i*_ represents the unobserved factors associated with each prefecture-level city that does not vary over time; *ε*_*it*_ is white noise, representing random error terms that obey a standard normal distribution.

Next, to systematically study the nonlinear effects of digital financial development on income distribution in China, we introduce the "threshold regression" method proposed by Hansen (1999) to establish a single panel threshold model and a double panel threshold model of digital financial development and income distribution.


$$GINI_{it}=\beta_{0}+\beta_{1}*DE_{it}I\left(q_{it}\leq \gamma \right)+\beta_{2}*DE_{it}I\left(q_{it}>\gamma \right)+\beta_{3}CV_{it}+\mu_{i}+\varepsilon_{it}$$ (4)

$$\begin{array}{l} GINI_{it}=\beta_{0}+\beta_{1}*DE_{it}I\left(q_{it}\leq \gamma_{1}\right)+\beta_{2}*DE_{it}I\left(\gamma_{1}<q_{it}\leq \gamma_{2}\right)+\\ \beta_{3}*DE_{it}I\left(q_{it}>\gamma_{2}\right)+\beta_{4}CV_{it}+\mu_{i}+\varepsilon_{it} \end{array}$$ (5)

Where: *GIN* denotes the Gini coefficient; *DE* denotes the level of development of regional digital finance; *I*(*q*_*it*_ ≤ *γ*) is the indicative function; *γ* is the threshold value of the single panel threshold model; *γ*_1_, *γ*_2_ is the first threshold value and the second threshold value of the double panel threshold model, respectively; the remaining control variables are consistent with the previous section of the article.

## IV. Results and discussion

### 1. Baseline regression results

In this paper, the regression Eq (3) is estimated by mixed model (Pooled), random effect model (RE), and fixed effect model (FE), respectively. Here, to make the coefficients more intuitive, we divide the digital financial development level index (DE) by 100, adjust it to a benchmark variable of 1, and multiply the Gini coefficient by 100. The regression results are shown in Table 2. Here, the level of digital financial development (DE) is taken as the core explanatory variable, and (1)-(3) are the estimated results obtained by including a series of control variables, and the primary focus here is on the effect of the core variable—the level of digital financial development (DE).

**Table 2:** Estimation results of the baseline model.

	(1)	(2)	(3)
	Pooled	RE	FE
DE (Digital Financial Development Level)	2.273***	2.273***	1.885***
	(16.83)	(16.83)	(10.93)
Rgdp (GDP per capita)	0.291***	0.291***	0.291***
	(3.62)	(3.62)	(3.29)
rgdpsq (squared GDP per capita)	-0.00485***	-0.00485***	-0.00463***
	(-2.90)	(-2.90)	(-2.62)
Fdi (foreign direct investment)	-0.637**	-0.637**	-0.625*
	(-2.06)	(-2.06)	(-1.94)
open (level of foreign openness)	-0.100	-0.100	-0.0758
	(-0.36)	(-0.36)	(-0.25)
urb (level of urbanization)	0.0571***	0.057**	0.154***
	(3.18)	(3.18)	(5.60)
User (Internet penetration rate)	-0.385	-0.385	-0.454
	(-1.17)	(-1.17)	(-1.25)
FD (level of financial development)	0.185**	0.185***	0.223**
edu (level of education)	(2.34)	(2.34)	(2.63)
	-8.931**	-8.93**	-9.975**
constants	(-2.10)	(-2.10)	(-2.04)
	40.5**	40.57**	35.94**
sample size	(41.66)	(41.66)	(24.14)
	4900	4900	4900
R^2^	0.371	0.371	0.376
R^2^ (after adjustment)	0.269	0.269	0.277

Note: Data in parentheses are t-statistics,

*p<0.1,

**p<0.05,

***p<0.01.

For choosing the panel data model for estimation, we first calculate the statistic to test whether to choose the fixed effects model or the random-effects model. Hausman’s test value is 26.10, and the corresponding p-value is 0.001, which rejects the original hypothesis. Thus, the fixed effects model is chosen, so column (3) results are more reliable. The estimated value of the coefficient of DE here is 1.885, which means that, on average, a one-unit increase in the digital financial development index, controlling for other variables, will expand social income inequality as measured by the Gini coefficient by 0.0001885 units. Among the control variables, the coefficients of the rgdp and rgdpsq variables are one positive and one negative, the urb and FD variables are significantly positive, the FDI and Edu variables are significantly negative, and the coefficients of the open and user variables are negative, but not significant.

According to the estimation results of the basic model, it can be seen that there is a more significant positive relationship between digital financial development and income disparity, and digital financial development is a major influencing factor for the widening of the wealth gap in China in recent years. The results of the empirical analysis are in line with the current concerns in China about the digital divide arising in the digital economy, where the development of digital finance over the past decade has not narrowed the current income gap in China but instead has worsened the wealth gap in China. The small amount of literature currently has pointed out the relevant impact of digital financial development on income distribution. However, little literature has examined it from the perspective of the overall income distribution gap. This paper confirms the close relationship between the two using prefecture-level city data in China, similar to the findings of the currently existing studies on digital finance widening the urban-rural income gap.

### 2. Kuznets model

At different stages of social development, the development of digital finance presents a differentiated impact on the income distribution of the population. At the primary stage of digital financial development, not everyone can afford to pay for computers and Internet devices such as cell phones due to the limitations of socio-economic development. The first to use Internet technology and participate in digital finance is a group of wealthy people. The poor cannot access digital financial services because they cannot pay for essential equipment. Therefore, there is a wealth threshold for participation in digital finance. The rich do not lack the cost of paying for electronic devices and thus can enjoy digital financial services without a threshold, often quickly obtaining higher returns from digital finance. The continuous development of digital finance further relaxes the credit constraints of the wealthy. It increases the rate of return that the wealthy can obtain by renting out their capital, further advancing the wealth accumulation of the wealthy.

In many cases, the poor cannot start and invest freely due to financial constraints, thus limiting their capital gains. In the initial stage of digital finance development, the advancement of Internet technology will increase total social wealth. However, this benefit is more favorable to the wealthy class, and the increased wealth is less in the pockets of the poor, so the income gap is widened. As economic development continues to improve, digital finance development is further improved, and the barriers to entry are gradually lowered; the poor also have access to the Internet and digital financial services. Thus the credit constraints of the poor are relaxed more than before, and they can use more funds for entrepreneurship, which slows down the income gap with the wealthy class.

In general, in the initial development stage of digital finance, the income inequality within the region will deteriorate, and digital finance hurts the regulation of income distribution within the region. In contrast, only when digital finance rises to a particular stage within the region, its subsequent development will help adjust the income distribution gap within. Based on the above analysis, we introduce the quadratic term DEsq of digital finance development and construct the Kuznets model about digital finance development and income distribution of residents. The model is set as follows.


$$GINI_{it}=\alpha_{0}+\alpha_{1}*DE_{it}+\alpha_{2}*DE_{it}sq+\alpha_{3}*CV_{it}+\mu_{i}+\varepsilon_{it}$$

Here, the primary term *DE*_*it*_ and the secondary term *DE*_*it*_*sq* of digital financial development are included in the regression equation, and the bell-shaped relationship between digital financial development and income distribution is verified if *α*_1_ > 0 and *α*_2_ < 0 held simultaneously.

In order to investigate whether the effect of digital financial development on income distribution shows a Kuznets effect, we estimate the relationship using a mixed regression model (Pooled), a random-effects model (RE), and a fixed-effects model (FE), respectively, using the same F-test and Hausman test, and conclude that a fixed-effects model should be chosen.

The focus here is on the impact of digital financial development on income disparity, from the regression results in Table 3 that the primary term coefficient of DE is significantly positive and the second term coefficient is significantly negative, confirming the bell-shaped effect of digital financial development on the income gap presented by residents. With the continuous development of digital finance, income inequality within regions shows a phenomenon of deterioration before improvement. In addition, the direction of other control variables on income distribution is consistent with the estimation results in the previous chapter. For example, the Kuznets effect exists between economic development and income distribution; foreign direct investment can effectively alleviate the current problem of excessive income disparity; the increasing level of urbanization and financial development will worsen the current status of the income distribution. The reason for the less significant effect of education level on income distribution may be that the proportion of primary and secondary school students in a region is not a good proxy for the education level in the region, and I have not found a more suitable proxy variable due to my limited academic level.

**Table 3:** Estimation results of the nonlinear model.

	(1)	(2)	(3)
	Pooled	RE	FE
DE (Digital Financial Development Level)	3.827***	3.837***	3.279***
	(8.33)	(8.33)	(6.74)
DEsq (Digital financial development level squared)	-0.497***	-0.497***	-0.433***
	(-3.55)	(-3.55)	(-3.06)
Constant	39.25***	39.25***	35.03***
	(37.74)	(37.74)	(23.12)
Number of samples	4105	4105	4105
R^2^	0.375	0.375	0.979
R^2^ (after adjustment)	0.276	0.276	0.281

Note: Data in parentheses are t-statistics,

*p<0.1,

**p<0.05,

***p<0.01.

In addition, this paper calculates the inflection point of the bell curve, whose value is about DE = 390, which corresponds to the practical implication that the development of digital finance and income inequality are positively correlated in prefectures where the depth of digital finance usage index is below 390. In contrast, the development of digital finance and income inequality are negatively correlated in prefectures where the depth of digital finance usage index is above 390. The study shows that during the sample period, only Shanghai’s Digital Financial Depth of Use Index exceeds 390. Thus, most regions in China are still on the left side of the bell curve, i.e., the income gap will further widen with the development of the digital economy. At present, China is still quite far from crossing the inflection point of the bell curve, and there is still considerable room for effort.

### 3. Robustness test

The explanatory variables in the model can be correlated with the stochastic disturbance term, thus creating an endogeneity problem, which may lead to serious bias in the study results at this point. Therefore we need to correct the endogeneity problem. Here we use the lagged one-period LDE of the endogenous explanatory variable DE as an instrumental variable to replace the current period for re-estimation, and Table 4 shows the estimation results of each regression equation. The regression results show that the coefficient of the primary term of the lagged period of the level of digital financial development is positive and the coefficient of the quadratic term of the lagged period is negative and significant at the 1% significance level, and the signs of the coefficients of the remaining control variables are basically consistent with the above, indicating that the conclusions obtained from the above model estimation are robust to the existence of the Kuznets effect on the impact of digital financial development on the distribution of residents’ income.

**Table 4:** Robustness test results.

Lagged period of the variable	(1)	(2)	(3)
	Pooled	RE	FE
LDE (level of digital financial development)	4.000***	4.000***	3.559***
	(8.17)	(8.17)	(6.67)
LDEsq (square of the level of digital finance development)	-0.620***	-0.620***	-0.565***
	(-4.12)	(-4.12)	(-3.69)
Constant	41.35***	41.35***	38.13***
	(37.18)	(37.18)	(22.67)
Sample size	4105	4105	4105
R^2^	0.320	0.320	0.322
R^2^ (after adjustment)	0.191	0.191	0.195

Note: Data in parentheses are t-statistics,

*p<0.1,

**p<0.05,

***p<0.01.

## V. Analysis of the threshold effect of digital finance development

### 1. Threshold variable description

We use economic development as a threshold variable to verify the threshold effect of digital financial development on income distribution in China, which is discussed in Greenwood’s (1990) classic paper on the differential effects of financial development on income distribution under different economic development conditions. Thus this paper extends to the effect of digital financial development on income distribution. This paper extends to the effect of digital financial development on income distribution. The participation of digital finance requires both specific electronic devices and certain Internet knowledge, so the effect of digital finance development on income distribution is different in different regions under different levels of economic development. When the local economy is relatively backward, only a few wealthy people can obtain mobile Internet devices to successfully participate in various activities of digital finance and generate investment income.

In contrast, most people are excluded from the threshold of digital finance, which will further widen the income gap. As society continues to develop and people’s income levels continue to rise, more and more people have access to mobile devices and basic Internet knowledge, digital finance is rapidly expanding among the population, and people are participating in various activities of digital finance, widening their income channels. As a result, the income gap within the region will continue to narrow. Currently, the Internet infrastructure in many poor areas is not well developed, and the lack of Internet base stations hinders residents’ access to the Internet. However, with the continuous development of the economy, the infrastructure in urban and rural areas becomes more and more complete, and more residents can enjoy the convenience and benefits brought by Internet finance without any obstacles. The poor people can thus achieve higher income and escape from poverty. The above analysis shows that economic development within a region is an essential factor affecting the distribution effect of digital finance.

Simply put, when the level of regional economic development is not high, and the penetration rate of digital finance is also low, it is often the rich with more initial wealth who participate in digital finance, further strengthening their wealth accumulation. At the same time, the poor are forced to block from the threshold of digital finance because of their initial wealth or the lack of knowledge reserves, and the income gap then widens. The Internet infrastructure will be laid out perfectly when the regional economic level develops to a particular stage. The residents in the region will no longer be excluded from the digital financial market. Therefore, digital financial development will have a different impact on the income distribution of residents than the former.

### 2. Results and discussion

Following the idea of [43], we first test whether the model has a threshold effect and determine the particular form of this threshold model. Based on the classical literature, the regional economic development level (GDP) is set as the threshold variable in this paper. Table 5 reports the F-values and P-values obtained by testing the threshold effect under the single and double threshold hypotheses.

**Table 5:** Threshold effect test.

Model	F-value	P-value	1% critical value	5% critical value	10% critical value
Single Threshold	86.01***	0.007	93.885	68.094	57.619
Double threshold	62.47*	0.100	96.333	71.996	62.807
Triple Threshold	22.64	0.857	107.976	82.863	69.551

Table 5 shows that the single threshold model passes the significance test with a value of 86.01 and a value of 0.007, so the original hypothesis of the linear model is rejected; continuing to search for the second threshold, the double threshold effect also passes the significance test with a value of 62.47 and a value of 0.100 at a confidence level of 0.1; then searching for the third threshold, the triple threshold effect is found to be insignificant, so the original hypothesis of the double threshold model is accepted. Thus, the above empirical results verify the nonlinear effect presented by digital financial development on income distribution, and the effect presents a double threshold feature.

Next, other threshold estimates were done for the sample, and the results are shown in Table 6, where the first threshold was calculated to be $3.1722 thousand with a 95% confidence interval of [3.1640,3.1844]; the second threshold was $10.344 thousand with a 95% confidence interval of [10.2648,10.3757].

**Table 6:** Threshold estimates and confidence intervals.

	Estimated value	95% confidence interval
First threshold	3.1722	[3.1640,3.1844]
Second threshold	10.3444	[10.2648,10.3757]

The estimation results of the threshold model are reported in Table 7. The results show a significant nonlinear characteristic of the impact of digital financial development on income distribution. We divide three major income blocks by the threshold variable, GDP per capita, corresponding to the calculated threshold value, and focus on the differences in the impact of digital financial development on income distribution under regions with different levels of economic development. The independent variable of the threshold model is digital financial development, and the dependent variable is the resident income gap. From the previous discussion, we can see that digital financial development significantly widens the resident income gap, so the regression coefficient of digital financial development is positive, and the larger this coefficient indicates that the negative effect of digital financial development on resident income distribution is greater, and the resident income gap is larger. The regression results show that when the per capita income does not exceed the first threshold value of $3,172.2, the impact coefficient of digital financial development is 1.743 and is significant at the 1% level; when the per capita income exceeds the first threshold value of $3,172.2 but is lower than the second threshold value of $10,344, the estimated coefficient of digital financial development increases from 1.743 to 2.329, indicating that when the regional income level rises to this region, the negative effect of digital financial development on income distribution increases; when the per capita income crosses the second threshold value of $10,344, the coefficient of digital financial development on income distribution decreases to 2.094, indicating that the negative effect of digital financial development on income distribution will weaken when the regional economic development level increases to a specific interval. The negative effect of digital financial development on the income distribution of residents in the region is the largest when the economic development level is located in the interval of $3172.2 to $10,344. Thus, this paper concludes that the effect of digital financial development on the income distribution of residents is closely linked to the economic development level of its location, and digital financial development at different economic levels has different effects on the income gap within the region showing a significant threshold feature.

**Table 7:** Regression results of the threshold panel model.

Variables	Coefficient estimates	Standard deviation	t-value	P-value	95% confidence interval
*DE*_*it*_ · *I* (*q*_*it*_ ≤ *γ*_1_)	1.743	0.204	8.53	0	[1.342,2.143]
*DE*_*it*_ · *I* (*γ*_1_ < *q*_*it*_ ≤ *γ*_2_)	2.329	0.196	11.86	0	[1.944,2.714]
*DE*_*it*_ · *I* (*q*_*it*_ > *γ*_2_)	2.094	0.158	13.26	0	[1.784,2.404]

In conclusion, under the conditions of low economic development level, digital financial development will widen the income gap within regions, but the impact effect is weak in this period; as the economic development level further increases, the negative effect of digital finance on income distribution will increase; and when the economic development level rises to a particular stage, the negative effect of digital financial development on income distribution begins to weaken. As a whole, the impact coefficient of digital finance development in each region is positive, proving that digital finance development is an essential reason for the current deterioration of income distribution in China, which is precisely consistent with the findings in the previous section of this paper.

Due to the unevenness of China’s regional economic development, there are more significant differences in the impact of digital financial development in each region. In order to more intuitively grasp the overall impact of digital financial development on the level of income distribution in China’s regions, the threshold variables are used here as criteria to further divide the sample. According to the corresponding division of China’s regions in Table 8 above, more than half of China’s prefecture-level cities had real per capita incomes below RMB 3,172.2 in 2011. However, from 2012 to 2018, most prefecture-level cities had per capita incomes in the range of RMB 3,172.2 to RMB 10,344, i.e., the most extensive range in which the effect of digital financial development widens income disparity, while per capita incomes above RMB 10,344 prefecture-level cities are always in the minority, but there is a rising trend in recent years. Therefore, prefecture-level cities with different levels of economic development can adopt different digital economy development policies, and further economic development can effectively curb the negative impact of digital financial development on residents’ income distribution.

**Table 8:** 2011–2018 Number of prefecture-level cities by threshold.

Year	rgdp≤3172.2	3172.2<rgdp≤10344	rgdp>10344
2011	161	100	6
2012	128	138	7
2013	112	156	10
2014	113	157	27
2015	82	180	17
2016	75	180	19
2017	67	211	20
2018	27	219	46
2009	41	202	37
2020	25	213	42

## VI. Conclusions and recommendations

A significant question to be addressed in this paper is whether the increase in social wealth from digital financial development is distributed equally to the entire population, or is it more skewed toward the rich or the poor? A multi-stage empirical study is conducted using data on prefecture-level cities and various econometric models. First, we collect the primary data on digital finance development and use the statistical yearbook data of each prefecture-level city to measure the Gini coefficient of each region, which is used as the primary data work for the empirical evidence. In terms of econometric tests, firstly, a linear panel model is used to examine the impact of digital financial development on the distribution of residents’ income. The article finds that it has a positive impact on the income distribution gap. Then, based on the classical G-J model theory, the paper further examines the Kuznets effect presented by digital financial development and resident income distribution and finds that there is indeed a bell-shaped curve. Finally, the article also uses the threshold model to test the threshold effect on the impact of digital financial development on income distribution in different economic development regions, and it can be seen that its effect on income distribution under different economic development level regions is different, and there is indeed a double threshold effect. The main conclusions of this paper are as follows.

First, with the comprehensive promotion of digitalization and the increasing coverage of mobile Internet, China’s digital finance development level has increased significantly. However, the degree of perfection of the digital finance market does not match the degree of development of the digital product market. While China’s Internet technology has made world-renowned achievements, the expansion of China’s digital financial volume has been very rapid, and digitalization has been fully applied at all levels, setting off a wave of reform in various industries. However, there are still significant development inequalities in the digital finance market, as different people do not face the same digital services. There are still significant differences in digital finance development between and within regions, with digital finance development in eastern coastal cities significantly better than in central and western regions. However, this gap is narrowing as China’s overall market continues to develop and improve. Second, the widening effect of digital financial development on income inequality is a significant impediment to improving the income distribution gap in China today. The article finds that digital financial development is positively correlated with income disparity through a linear panel empirical test of 280 prefecture-level cities and uses the digital indicator of financial inclusion to replace the level of digital financial development. The results do not differ significantly, further confirming the assertion that the higher the level of digital financial development, the greater the income disparity among residents, indicating that the increase in the level of digital financial development in China during the sample period is a This suggests that the increase in the level of digital financial development in China during the sample period is an essential factor influencing the widening of the income distribution gap. Third, there is a Kuznets effect on the impact of digital financial development on the income distribution of residents in China. The differences influence the effect of digital financial development on income distribution in the level of economic development within regions with significant threshold characteristics. There is a bell-shaped curve relationship between the degree of digital financial development measured by the depth of digital financial use and the Gini coefficient of prefecture-level cities, indicating that as the level of digital financial development continues to increase, the income gap within regions in China shows a trend of change that first increases and then decreases.

On the whole, the development of digital finance in China is not yet perfect, and most of the prefecture-level cities have not yet crossed the inflection point of the bell curve but are still in the upward range on the left side of the curve. The impact of digital finance development on income disparity shows a significant positive correlation, and the level of income inequality will further increase with the development of regional digital finance. In addition, the effect of digital financial development on income distribution is influenced by the level of economic development within the region. Regions at different stages of economic development have differential effects of their digital financial development on income distribution within the region and show non-monotonic bithreshold characteristics: its adverse effects first gradually increase with the rise of economic development level, and they tend to weaken. Thus, the impact of digital financial development on income distribution in each region of China is not an isolated process but works together and interacts with local socio-economic development.

Synthesizing the previous conclusions, we will get the following policy insights. First, to narrow the divide created by regions in the process of digital development at the source. Due to the unbalanced nature of China’s economic development, the social population has gradually opened up gaps in income and education, resulting in the differentiation of various classes. The inequality in income and education will lead to gaps in access to information and processing of information among different groups in different regions in the current wave of informationization, thus creating the digital divide phenomenon. To solve the current digital divide, the focus is to vigorously promote social equity, focus on solving the problem of uneven development among various regions and groups, and pay more attention to education in poor areas in the central and western regions and rural areas, actively carry out policy promotion, attract educational resources to enter, improve the level of the human capital of groups in less developed regions, and enhance the ability of residents in the region to have equal access to information technology. Second, accelerate the digitalization process and coordinate policies related to opening up to the outside world, urbanization, Internet penetration, and financial development so that policies are biased toward less developed regions and increase their information dividends. Each region should speed up the local information construction and give better play to the advantages of Internet technology so that the development of local digital finance can achieve the inflection point crossing as early as possible and reduce the income gap within the region. Third, take advantage of the matching and complementary relationship between digital financial development and other economic systems to effectively play a positive role of digital technology in improving income distribution. As mentioned in the text, the impact of digital financial development on intra-regional income distribution is not isolated. The human capital and physical capital possessed by individuals will also show interactive effects and digital financial development. Therefore, when formulating policies on digital finance development, government departments should not only regard digital finance as an independent variable affecting regional income distribution but also ignore the correlation with other development factors. The main aspects are as follows: (1) to improve the level of local economic development and further increase the income level of residents; (2) to promote the regional balance of financial expenditure on education, to promote the development of education in a balanced direction and to achieve equity in educational opportunities at all levels; (3) to continue to attract foreign investment and optimize the trade structure, to promote the flow of financial expenditure to less developed regions and disadvantaged groups. All of the above measures can promote the development of China’s income distribution in the direction of equity.

This paper provides some theoretical basis and data support on how to better play the current system of digital finance, optimize income distribution, and promote the role of digital technology in financial inclusion. However, there are still some shortcomings in this paper, which can be the direction of subsequent research.

First, the realistic explanation about the nonlinear characteristics related to the impact of digital financial development on income distribution needs to be further improved. The focus of this paper is to explore the nonlinear relationship between digital financial development and income distribution, and it is found that the impact of digital financial development on the income distribution of residents shows significant Kuznets effect as well as nonlinear double threshold characteristics. However, the article’s explanation of the above measurement results is rather thin, so the follow-up should continue to use theoretical combined with empirical methods to more systematically study the current income distribution under the digital financial system and further dig deeper on the basis of existing studies.

Second, the measurement of the development level of digital finance in this paper is limited to the data of the digital inclusive finance system built by Peking University, and there is no timely measurement of some new businesses that have changed, which is not enough to reflect the real level of the current development of digital finance in China. In future research, if possible, we will construct our own indicators on the development of digital finance in China, aiming to more accurately describe the current state of digital finance development.

## Supporting information

S1 Dataset (XLSX)
